# Hospital Outbreaks of Middle East Respiratory Syndrome, Daejeon, South Korea, 2015

**DOI:** 10.3201/eid2306.160120

**Published:** 2017-06

**Authors:** Jung Wan Park, Keon Joo Lee, Kang Hyoung Lee, Sang Hyup Lee, Jung Rae Cho, Jin Won Mo, Soo Young Choi, Geun Yong Kwon, Ji-Yeon Shin, Jee Young Hong, Jin Kim, Mi-Yeon Yeon, Jong Seok Oh, Hae-Sung Nam

**Affiliations:** Korea Centers for Disease Control and Prevention, Cheongju, South Korea (J.W. Park, K.J. Lee);; Sokcho Medical Center, Sokcho, South Korea (K.H. Lee);; Ulsan Metropolitan City Hall, Ulsan, South Korea (S.H. Lee);; Daejeon Provincial Government, Daejeon, South Korea (J.R. Cho);; Chuncheongbukdo Provincial Government, Cheongju (J.W. Mo);; DaeChung Hospital, Daejeon (S.Y. Choi);; Ministry of Health and Welfare, Sejong, South Korea (G.Y. Kwon);; Eulji University School of Medicine, Daejeon (J.-Y. Shin);; Konyang University College of Medicine, Daejeon (J.Y. Hong);; Chungnam National University School of Medicine, Daejeon (J. Kim, M.-Y. Yeon, H.-S. Nam);; Gwanggyo Ean Pediatric Clinic, Suwon, South Korea (J.S. Oh)

**Keywords:** Middle East respiratory syndrome, MERS, hospital-acquired condition, outbreaks, viruses, zoonoses, South Korea

## Abstract

From May through July 2015, a total of 26 cases of Middle East Respiratory Syndrome were reported from 2 hospitals in Daejeon, South Korea, including 1 index case and 25 new cases. We examined the epidemiologic features of these cases and found an estimated median incubation period of 6.1 days (8.8 days in hospital A and 4.6 days in hospital B). The overall attack rate was 3.7% (4.7% in hospital A and 3.0% in hospital B), and the attack rates among inpatients and caregivers in the same ward were 12.3% and 22.5%, respectively. The overall case-fatality rate was 44.0% (28.6% in hospital A and 63.6% in hospital B). The use of cohort quarantine may have played a role in preventing community spread, but additional transmission occurred among members of the hospital cohort quarantined together. Caregivers may have contributed in part to the transmission.

A few respiratory viruses constitute emerging threats to global health security (*1*); among them are Middle East respiratory syndrome (MERS) coronavirus (MERS-CoV), which has caused outbreaks in Saudi Arabia (*2*,*3*). The major MERS outbreaks that occurred during 2012–2015 have been in or near the Arabian Peninsula. However, information on the epidemiologic features of MERS is insufficient, especially for different environmental and cultural settings. The 2015 MERS outbreak in South Korea could provide more information about the epidemiology of MERS because it was the largest outbreak outside the Middle East (*4*).

The first case of MERS in South Korea was reported on May 20, 2015. The patient had flown among several countries in the Middle East (Bahrein, the United Arab Emirates, Saudi Arabia, and Qatar) and became the source of consecutive hospital-to-hospital transmissions after his return to South Korea, which led to 186 laboratory-confirmed cases (*5*) and 38 deaths. Hospital-to-hospital transmission involved 17 hospitals and originated from 1 hospital (hospital P) (*5*,*6*). This transmission was attributable to “hospital shopping” by some MERS patients (*4*,*5*) and was particularly evident in Daejeon, which is the fifth largest city in South Korea. The index case-patient for nosocomial transmission in Daejeon had initially traveled from his home city of Daejeon to Pyeongtaek, South Korea, seeking healthcare at hospital P, after which he returned to Daejeon. Subsequently, 2 hospitals in Daejeon experienced MERS cases attributable to this patient. This index case-patient in Daejeon was consecutively hospitalized at hospital A in Daejeon during May 22–28, 2015, and at hospital B during May 28–30, 2015. Thereafter, an additional 25 MERS cases (14 in hospital A, 11 in hospital B) were reported.

After the South Korea government recognized the outbreak of MERS in Daejeon, cohort quarantine (isolation of persons who had been in contact with patients with confirmed cases in the hospital ward) was applied. This quarantine seems to have played a useful role in preventing the spread of MERS-CoV to the local community. We describe the MERS case-patients, the epidemiologic features of the disease, and the quarantine policy used to prevent additional transmission.

## Methods

### Setting

Hospital A is a 300-bed general hospital in Daejeon. The outbreak occurred in ward 51 on the fifth floor, where 13 rooms (5 with 7 beds, 6 with 4 beds, 1 with 2 beds, and 1 with 1 bed) are located. Hospital B is an 800-bed university hospital in Daejeon. The main outbreak occurred in ward 101 on the ninth floor, where 16 rooms (7 with 6 beds, 1 with 4 beds, 1 with 2 beds, and 7 with 1 bed) are located.

### Data Collection and Exposure Assessment

Epidemiologic investigators of the Korea Centers for Disease Control and Prevention started their outbreak investigation with face-to-face interviews of the index case-patient in Daejeon and the 25 additional case-patients with confirmed MERS-CoV infection. We collected data on the demographic characteristics and the clinical, contact, and MERS-CoV exposure histories and thoroughly reviewed the medical records of the case-patients to identify symptoms, underlying concurrent medical conditions, laboratory findings, and clinical courses of illness. Clinical outcome was classified as recovery or death, and the ambulation status of the inpatients at the time of admission was clarified.

We collected the names of inpatients, their room numbers, medical staff, and caregivers (family members or professionals hired by the family or hospital) exposed to MERS-CoV in each hospital. The duration and route of exposure were further determined by reviewing recordings from closed-circuit televisions placed in the hospitals. Moreover, we used the floor plan of each hospital to estimate the spatial distributions and transmission routes of the virus within the hospitals. These estimates enabled us to identify a possible location of exposure and a transmission route for each confirmed case. When a patient with a confirmed case had experienced several possible exposures, we determined the most probable exposure by author consensus. Persons who had had face-to-face contact with patients with confirmed cases were considered the closest contacts. When the data were ambiguous, the following were reviewed independently by the Korea Centers for Disease Control and Prevention and the Daejeon In-Depth team: all potential exposures by symptom onset; disease duration; physical distance from a patient with a confirmed case; and infector factors including ambulation status, symptoms (including a productive cough), and sharing of caregivers. When a patient with a confirmed case had been subjected to several potential exposures, the most probable exposure was determined by consensus of the 2 teams. An expert member of the Korean Society of Epidemiology reviewed all decisions. The process was repeated until a final consensus was obtained.

### Laboratory Diagnoses

Sputum samples from the persons suspected of having MERS were collected in sterile cups and sent to qualified local or national laboratories for confirmation. As a confirmatory test, a real-time reverse transcription PCR of nucleic acid extracted from sputum specimens was performed (*5*). Cycle threshold values were also measured to quantify viral loads. For each patient with a confirmed case, the Korea Centers for Disease Control and Prevention assigned a case number according to the order of confirmation during the 2015 MERS outbreak in South Korea. For example, the case number of the index case-patient in Daejeon was 16.

### Data and Statistical Analyses

The cases were described in case-series form. Attack rates were calculated as the number of cases per number of exposed persons (defined as persons who had experienced face-to-face contact with a symptomatic MERS case-patient in either hospital or as persons who had been in the same hospital ward as the symptomatic case-patients). Such persons were identified from the outbreak investigation reports and the lists of those undergoing cohort or home quarantine. To assess differences in attack rates and case-fatality rates according to independent variables, we performed χ^2^ and Fisher exact tests by using SAS software version 9.3 (SAS Institute, Inc., Cary, NC, USA). Comparisons were considered significant at p<0.05 and marginally significant at p<0.1 (both p values were 2-tailed).

We defined the incubation period as the time from exposure to onset of MERS-associated symptoms, including nonspecific signs and symptoms such as fever, chills, cough, sore throat, sputum production, dyspnea, myalgia, headache, nausea, vomiting, diarrhea, and abdominal discomfort. If the exposure period was >2 days, a single interval-censored estimate of the incubation period was computed by using the earliest and latest dates of exposure and the date of symptom onset for each case-patient (coarseDataTools package in R statistical software version 3.2.2) (*7*). To construct cumulative fraction curves of all cases by incubation period, we calculated the log-normal density function by fitting the interval-censored data on incubation periods. To do this, we used the maximum-likelihood method and calculated the medians and 5th and 95th percentiles of the incubation periods.

## Results

### Description of the Daejeon Index Case-Patient 

The Daejeon index case-patient (case-patient 16), a 41-year-old man, lived in Daejeon and was a former smoker (10 to 20 pack-years). He had undergone colon surgery in August 2014 at hospital P. The index case-patient of the MERS outbreak in South Korea (case-patient 1) was in hospital P during May 15–17, 2015. The Daejeon index case-patient was admitted to hospital P at the same time (May 15–18, 2015) for a follow-up colonoscopy. After discharge on May 20, the Daejeon index case-patient felt feverish and had chills, cough, general weakness, and diarrhea. Because of these symptoms, he was hospitalized in hospital A in Daejeon during May 22–28; the room was shared by 3 inpatients and 1 caregiver. Because his symptoms did not improve, he was transferred to the emergency department at hospital B. After hospitalization in ward 101 in hospital B, he was suspected of having MERS and was isolated in a negative-pressure room on May 30. Ultimately, he became the 16th confirmed MERS patient of 186 total case-patients during the 2015 outbreak.

Before the Daejeon case-patient was isolated, those around him did not use protective equipment. Therefore, virus was spread from him during his first 10 days of illness, before MERS diagnosis and isolation. When we checked the closed-circuit television recordings from hospital A to estimate how many persons could have been in contact with the Daejeon index case-patient, we found that he had been in several sections of the hospital ward, in particular those located on the left side of the nurse station. These sections included his admission room, a restroom, the nurse station, the foyer, and the hall in front of the elevators. The Daejeon index case-patient had potentially contacted every inpatient in the same hospital ward. Therefore, we classified all patients and caregivers in that ward as possible contacts.

### Description of Patients with Confirmed Cases

A total of 26 cases (including the index case) were confirmed in the 2 hospitals, and 11 case-patients died of MERS (4 in hospital A and 7 in hospital B) (Technical Appendix). Other than the index case, 14 cases occurred in hospital A and 11 in hospital B. Case-patients 30, 38, and 128 were admitted to the same room in hospital A as the Daejeon index case-patient. Case-patient 85 was a caregiver hired by case-patient 128, so she was in the same hospital room in hospital A during May 22–28. Case-patients 23, 24, 31, 36, and 95 were admitted to the same room in hospital B as the Daejeon index case-patient. Case-patient 82 was the wife of case-patient 36; case-patient 106 was a caregiver hired by case-patient 36; and case-patient 127 was the wife of case-patient 24. Therefore, when caregiving, they were in the same room as the Daejeon index case-patient.

The median age of the case-patients was 71 (interquartile range 38–86) years; 13 (52.0%) were male; 6 (24.0%) were commercial caregivers; and 3 (12.0%) were family caregivers. A total of 18 (72.0%) case-patients had underlying diseases; 7 (28.0%) had pulmonary diseases, such as asthma, chronic obstructive pulmonary disease, idiopathic pulmonary disease, lung cancer, and pulmonary tuberculosis.

All patients reported fever. Other signs and symptoms included chills (10 patients, 38.5%), cough (8, 30.8%), sputum (6, 23.1%), myalgia (9, 34.6%), headache (4, 15.4%), dyspnea (6, 23.1%), nausea (3, 11.5%), diarrhea (6, 23.1%), sore throat (3, 11.5%), rhinorrhea (2, 7.7%), hemoptysis (1, 3.8%), and abdominal discomfort (1, 3.8%)

### Quarantine

To prevent the spread of MERS-CoV to the local community, on June 1, 2015, the government of South Korea ordered cohort quarantine, which hospitals A and B followed (Table 1). Persons with a history of exposure to patients with confirmed MERS were isolated in the same hospital ward. 

**Table 1:** Quarantine policy to prevent additional transmission of MERS, Daejeon, South Korea*

Action
• The cohort quarantine applied to admitted patients and their caregivers (professional or family) exposed to the MERS case-patients.
• Inpatients admitted to the same hospital room before quarantine were quarantined in the same room because their degree of exposure was probably the same. Their caregivers were also quarantined in the same room because of the need for caregiving.
• The medical staff (physicians, nurses, and medical technologists) exposed to the MERS case-patients were subjected to home quarantine. However, members of the households of medical staff were not subjected to home quarantine until and unless that medical staff member exhibited any symptoms. Contact between household members and the medical staff member was severely restricted.
• The wards under cohort quarantine were controlled by unexposed medical staff using level D protectors (Microguard 2000; 3M, Bracknell, UK). Each protector included an N95 mask, protective glasses, a whole-body protective gown, gloves, and boots.
• The body temperature of persons (including inpatients and caregivers) and medical staff admitted to cohort or home quarantine was checked, and these persons were clinically interviewed twice daily. If they reported any symptoms (including a febrile sensation or chills) or if they were asymptomatic but with a body temperature >37.5C°, they were immediately placed in a quarantined area at each hospital. The KCDC performed laboratory tests at this stage; the results were available 3 d later. If the doctor in charge strongly suspected MERS, that patient could be transferred, with careful precautions, to a national isolation hospital within 1 d.
• All wards were disinfected by use of sodium hydrosulfite, 80% (vol/vol) alcohol, and 2% (vol/vol) chlorhexidine twice during each shift, thus 6 times/d.
• South Korea operates a nationwide medical insurance scheme; all costs incurred by MERS patients were covered.
• Persons with confirmed MERS were transferred to another quarantine room that had negative-pressure equipment.
Strategies for caregivers
• The infection control team carefully explained the risk for MERS and the need for cohort quarantine to all caregivers. Some caregivers did not wish to remain in hospital wards with inpatients. They were taken home and placed in in-home quarantine and used the same MERS quarantine strategy applicable to medical staff in close contact with the patients.
• Caregivers attended only noninfected inpatients who required total care. If an inpatient was confirmed to have MERS, nursing care was provided by professional nurses wearing protectors.
• The infection control team continuously educated caregivers on how MERS was transmitted and how to prevent infection. Caregivers were told to wear protectors (N95 masks, vinyl gowns, and gloves) and to not touch each other. However, during the first week of quarantine, checks of closed-circuit television footage showed that the protector and contact rules were sometimes not obeyed in hospital A.
• Hospital A designated 2 rooms for caregivers in the quarantine ward. The caregivers could use these rooms when they were not actively engaged in patient care.

*KCDC, Korea Centers for Disease Control and Prevention; MERS, Middle East respiratory syndrome.

### Epidemic Curve 

**Figure 1:**
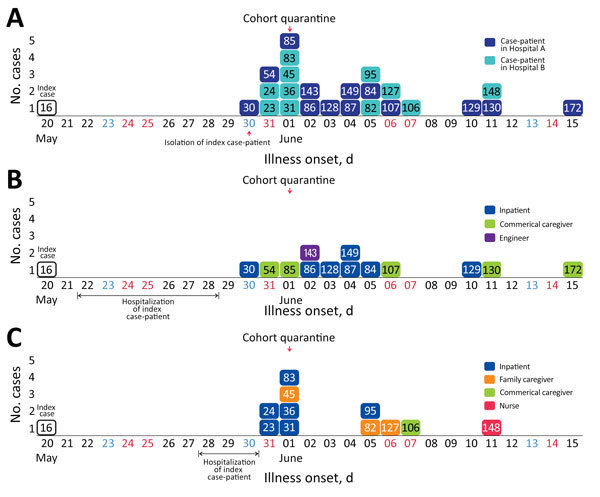
Epidemic curves for the Middle East respiratory syndrome outbreak in Daejeon, South Korea, 2015. The cases are numbered in the order in which they were confirmed in the context of all cases reported during the outbreak. A) Hospitals A and B; B) Hospital A; C) Hospital B. Case-patient 38 is not included because date of illness onset is unknown. Black, weekday; blue, Saturday; red, Sunday or holiday.

After the index case-patient in Daejeon spread MERS-CoV in Daejeon, the first case occurred on May 30, 2015, and the last on June 15, 2015 (total outbreak duration 17 days) (Figure 1). The epidemic curve for hospital A suggested a relatively sporadic pattern compared with that for hospital B. The peak in hospital B comprised mostly patients who shared a hospital room with the index case-patient. Most MERS cases appeared later in professional or family caregivers rather than in inpatients.

**Figure 2:**
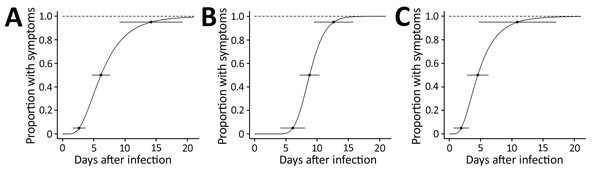
Estimated incubation periods for the Middle East respiratory syndrome outbreak in Daejeon, South Korea, 2015. Curves indicate estimated cumulative fractions of cases corresponding to the incubation periods, estimated by creating log-normal density functions fitting the observed data. Horizontal lines indicate 95% CIs for the 5th, 50th, and 95th percentiles of the estimated incubation periods. A) Total; estimated median incubation period was 6.1 (95% CI 4.7–7.5) days. B) Hospital A; estimated median incubation period was 8.8 (95% CI 7.2–10.4) days. C) Hospital B; estimated median incubation period was 4.6 (95% CI 2.9–6.2) days.

The estimated median incubation period for confirmed cases was 6.1 (95% CI 4.7–7.5) days (Figure 2). Incubation periods were 8.8 (95% CI 7.2–10.4) days for hospital A and 4.6 (95% CI 2.9–6.2) days for hospital B.

**Figure 3:**
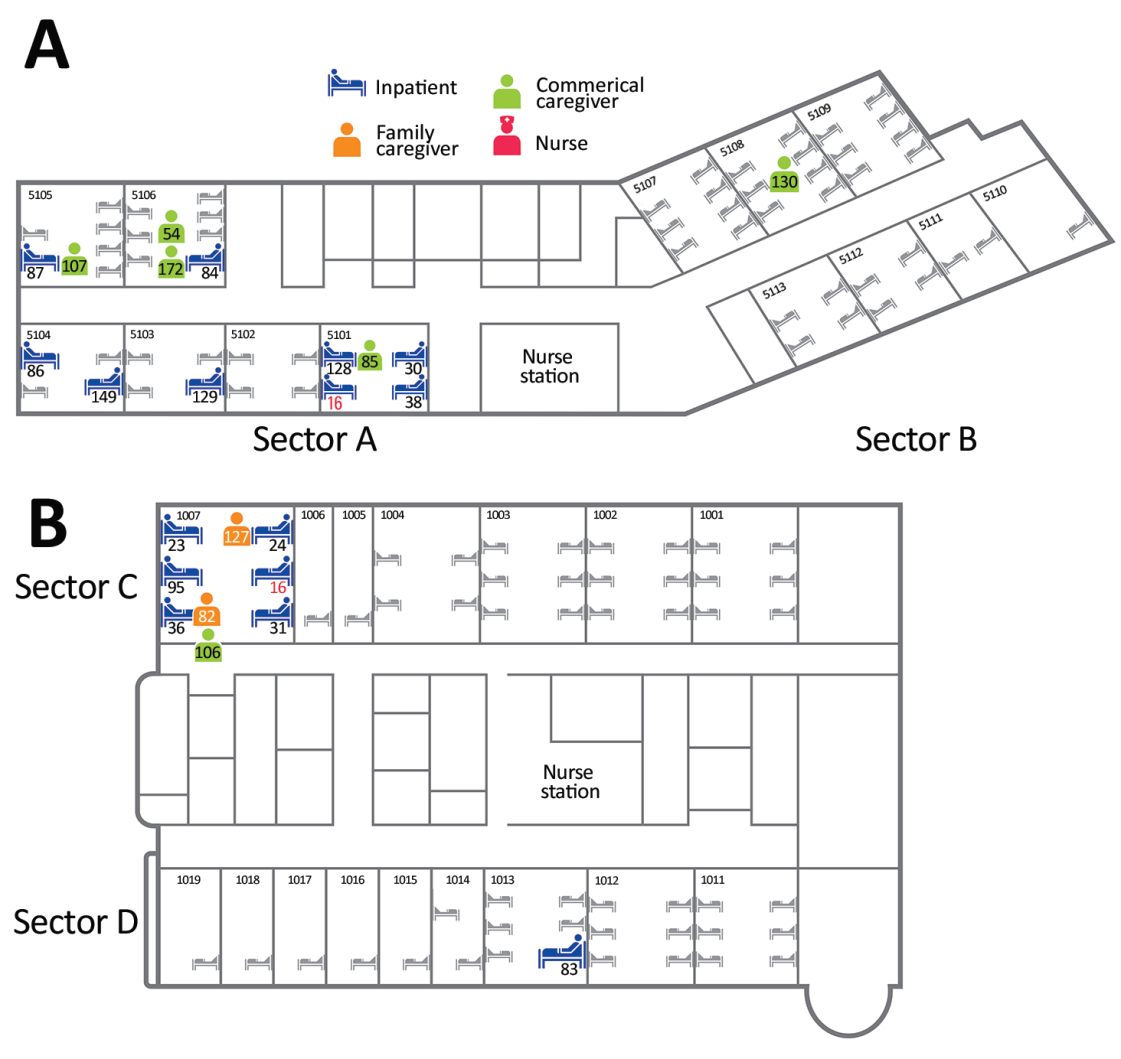
Locations of Middle East respiratory syndrome case-patients in hospitals A and B, Daejeon, South Korea, 2015, showing where case-patients were exposed to presumed infectors. Not shown are case-patient 143, an engineer working in hospital A, because the location of his exposure is unclear; case-patient 45, a family caregiver in either the emergency department or room 1015; and case-patient 148, a nurse in the intensive care unit.

In hospital A, the index case-patient was admitted to room 5101, in sector A (Figure 3). Thereafter, 12 case-patients were in sector A, and 1 was in sector B. However, the case-patient in sector B had a history of contact with case-patient 85, who was transferred to sector B from sector A for quarantine. Most case-patients were presumed to have been infected by the Daejeon index case-patient (case-patient 16). However, 3 instances of other transmission were noted: case-patient 85 to case-patient 130, case-patient 54 to case-patient 172, and several case-patients to case-patient 129. For this last instance of transmission, we could not identify the most probable source, because many possible exposures were evident (case-patients 54, 84, 86, 87, 107, and 149).

In hospital B, the index case-patient was admitted to room 1007, located on the upper side of ward 101 (sector C). Eight case-patients were in sector C. Case-patient 83 was in room 1013 on the opposite side of ward 101 (sector D). Case-patient 45 was in the emergency room and ward 101 with the index case-patient. Case-patient 148 was presumed to have been infected by case-patient 36 during performance of cardiopulmonary resuscitation in the intensive care unit.

### Attack Rate and Case-Fatality Rate

A total of 14 cases developed among 301 persons exposed in hospital A (attack rate 4.7%) and 11 among 371 persons exposed in hospital B (attack rate 3.0%) (Table 2). The attack rates for the sectors hosting the index case-patients (sector A of hospital A, sector C of hospital B; Figure 3) were higher than those for other sectors (sector B of hospital A, sector D of hospital B; Figure 3) (31.6% vs. 2.4% in hospital A, p<0.05; 18.2% vs. 6.5% in hospital B; Table 3). The probability of infection for a person admitted to the same rooms as the index case-patient was 75.0%. In both hospitals, attack rates were somewhat higher for caregivers (22.5%) than for inpatients (12.3%), although statistical significance was not attained.

**Table 2:** Middle East respiratory syndrome attack rates among all exposed persons, Daejeon, South Korea, 2015

Exposure	Hospital A			Hospital B			Total	
No. exposed /no. with confirmed case	Attack rate, %	No. exposed/no. with confirmed case	Attack rate, %	No. exposed/no. with confirmed case	Attack rate, %
Total	301/14	4.7		371/11	3.0		672/25	3.7
Inpatients	227/8	3.5		122/6	4.9		349/14	4.0
Same ward as index case-patient	62/8	12.9*		52/6	11.5*		114/14	12.3*
Other wards	165/0	0		70/0	0		235/0	0
Caregivers†	29/5	17.2*		32/4	12.5		61/9	14.8*
Same ward as index case-patient	17/5	29.4‡		23/4	17.4		40/9	22.5*
Other wards	12/0	0		9/0	0		21/0	0
Nurses	20/0	0		78/1	1.3		98/1	1.0
Doctors	8/0	0		35/0	0		43/0	0
Others§	17/1	5.9		104/0	0		121/1	0.8

*p<0.05 †Family or professional.  ‡p<0.1. §Paramedics, students, engineers, and visitors.

**Table 3:** Attack rates for Middle East respiratory syndrome among inpatients and caregivers in the same wards as the index case-patient, Daejeon, South Korea, 2015

Person	Hospital A			Hospital B			Total	
	No. exposed/no. with confirmed case	Attack rate, %		No. exposed/no. with confirmed case	Attack rate, %		No. exposed/no. with confirmed case	Attack rate, %
Total	79/13	16.5		75/10	13.3		154/23	14.9
Sex								
M	20/5	25.0		36/7	19.4		56/12	21.4*
F	59/8	13.6		39/3	7.7		98/11	11.2
Age, y								
30–64	28/5	17.9		28/3	10.7		56/8	14.3
>65	51/8	15.7		47/7	14.9		98/15	15.3
Role								
Inpatient	62/8	12.9		52/6	11.5		114/14	12.3
Caregiver†	17/5	29.4		23/4	17.4		40/9	22.5
Hospital or room								
Same sector‡	38/12	31.6§		44/8	18.2		82/20	24.4§
Same room	4/4	100¶		12/8	66.7¶		16/12	75.0¶
Other room	34/8	23.5		32/0	0		66/8	12.1
Different sector#	41/1	2.4		31/2	6.5		72/3	4.2
Ambulatory								
Yes	32/10	31.3		41/7	17.1		73/17	23.3
No	23/3	13.0		13/3	23.1		36/6	16.7
Not known	24/0	0		21/0	0		45/0	0

*p<0.1. †Family or professional. **‡**Sector A of hospital A or sector C of hospital B, as described in Figure 3. §p<0.05 when compared with attack rates in a different sector. ¶p<0.05 when compared with attack rates in other rooms. #Sector B of hospital A or sector D of hospital B (Figure 3).

The overall case-fatality rate was 44% (Table 4). This rate was higher for patients in hospital B (63.6%) than for those in hospital A (28.6%), although statistical significance was not attained.

**Table 4:** Case-fatality rates among all Middle East respiratory syndrome case-patients, Daejeon, South Korea, 2015

Concurrent condition	Hospital A			Hospital B			Total	
	No. incident cases/ no. fatal cases	Case-fatality rate, %		No. incident cases/ no. fatal cases	Case-fatality rate, %		No. incident cases/ no. fatal cases	Case-fatality rate, %
Total	14/4	28.6		11/7	63.6		25/11	44.0
Pulmonary	1/0	0		6/5	83.3		7/5	71.4
None or nonpulmonary	13/4	30.8		5/2	40		18/6	33.3

## Discussion

During the MERS outbreak in South Korea, 25 confirmed cases (including 11 deaths) occurred in Daejeon, all associated with the same index case-patient. Two hospitals were affected. The incubation periods and case-fatality rates for the 2 hospitals differed.

Under the South Korea healthcare system, patients can visit secondary hospitals and the emergency rooms of tertiary hospitals without limitation (*5*), which probably facilitated nosocomial transmission of MERS-CoV. Indeed, the outbreak in Daejeon accounted for 1 of the 3 major MERS-CoV outbreaks in South Korea. These observations underscored the importance of the outbreak in Daejeon, leading the South Korea government to focus resources on controlling transmission of the virus.

The estimated median incubation period for MERS during the outbreak we report was similar to that for outbreaks in the eastern province of Saudi Arabia in 2013 (*1*). Incubation period estimates may differ, depending on the method used to select exposure: the most probable exposure versus overlapping exposures. In our study, the incubation periods estimated by using both methods were similar. The incubation period estimated by using the most probable exposure method was 6.1 (95% CI 4.7–7.5) days, and that estimated by using the overlapping exposures method was 5.6 (95% CI 4.2–6.9) days. The overall attack rate among all exposed persons in Daejeon was similar to that for Pyeongtaek (*5*). The case-fatality rate of the outbreak in Daejeon was lower than that in the eastern province of Saudi Arabia in 2013 (*1*) but similar to that in Jeddah, Saudi Arabia, in 2014 (*3*).

Our results indicate various epidemiologic characteristics of MERS-CoV. All persons acquired infection in a hospital setting, which is consistent with the previous outbreak in Saudi Arabia, in which nosocomial spread was a major route of MERS-CoV transmission (*1*). The characteristics of the specific hospital seemed to affect attack rates and case-fatality rates. The index case-patient in Daejeon was consecutively admitted to 2 hospitals. The fifth floor of hospital A specializes in senile patients, most of whom have chronic illnesses, including Parkinson’s disease, paraplegia attributable to old infarctions, or amyotrophic lateral sclerosis. Most beds on the fifth floor are occupied by bedridden patients. The attack rate among caregivers was higher in hospital A than in hospital B. Because immobile patients require personal caregiving, their caregivers were required to be in prolonged close contact with patients, which might have resulted in a higher attack rate. Hospital B is a university hospital and thus contained more severely ill patients than hospital A. Ward 101, to which the Daejeon index case-patient was admitted, is the main ward of the pulmonary medicine department. We presumed that the case-fatality rate was higher for hospital B than hospital A because of underlying pulmonary disease, which has been reported to be a risk factor for development of more severe diseases (*8*).

Generally, cohort quarantine may be useful as an infection-control tool to limit virus transmission in hospitals in which susceptible inpatients are gathered or to more effectively detect infected patients (*9*). Hospitals A and B applied cohort quarantine. In this situation, cohort quarantine had several advantages and disadvantages. The primary purpose was to prevent the spread of MERS-CoV to the local community. After applying cohort quarantine, no further spread of MERS-CoV occurred other than within hospitals A and B. This result may have been achieved by quarantining all persons who had been in contact with MERS-CoV–infected patients and by refusing hospital entry to all susceptible persons. In addition, more cases were diagnosed promptly by active surveillance of the cohort. However, this policy had a limitation. One cohort accommodated inpatients and caregivers in the same hospital room; thus, if 1 person in the cohort was infected by MERS-CoV, others were exposed, increasing the probability of MERS-CoV transmission. This practice raises an ethical issue in terms of whether letting persons stay in the same room with potential MERS patients is justified by the purpose of preventing transmission of the virus to the community. Some caregivers at hospital A may have had difficulty complying with the quarantine policy (the protector and contact rules) because they cared for immobile patients. Thus, this practice may have increased transmission within the hospital.

We identified several cases with uncommon routes of transmission. Case-patient 148 was the head nurse of the intensive care unit to which case-patient 36 was admitted. When case-patient 36 experienced cardiac arrest, that nurse performed cardiopulmonary resuscitation while wearing a level D protector. However, afterward, she may have been exposed to MERS-CoV when she wiped sweat with her bare arm. Case-patient 143 was an employee of hospital A; he worked in information technology. He was employed by hospital A during January–May 30, 2015, and his bedroom was located on the seventh floor. His routine work routes, shown on closed-circuit television, did not reveal any close contact with case-patients; thus, the transmission route was unclear. We presume that he was infected by fomites in an elevator or exposed to a patient in a place lacking closed-circuit television coverage.

The transmission route for case-patient 83 was also unidentified. It is possible that some medical staff and caregiver, contaminated with MERS-CoV after visiting room 1007, may have visited case-patient 83 in room 1013. Of note, when case-patient 83 was exposed to MERS-CoV, the outbreak in Daejeon had not yet been recognized and hospital B had not yet implemented infection control strategies (e.g., handwashing; wearing gloves, masks, and vinyl gowns).

This study had several limitations. First, we cannot be certain that all chains of infection between case-patients have been identified. We did not perform serologic analyses to seek cases that were potentially missed; such missed cases may have affected the incubation period estimates and case-fatality rate. Second, closed-circuit television may not have captured all relevant movements.

In conclusion, in 2015, Daejeon experienced a hospital-associated outbreak of MERS-CoV. Two hospitals experienced nosocomial outbreaks, and virus transmission was evident among mostly inpatients and caregivers. To prevent the spread of the virus to the local community, we developed a unique and successful cohort quarantine policy. However, ethical issues associated with this policy require thorough discussion by policy makers. 

Technical Appendix Baseline characteristics of Middle East respiratory syndrome case-patients, Daejeon, South Korea, 2015.
