# Supplementary material for: Hospital Outbreaks of Middle East Respiratory Syndrome, Daejeon, South Korea, 2015
Source: Emerg Infect Dis. 2017 Jun;23(6):898–905. doi: 10.3201/eid2306.160120 (PMC5443424; doi:10.3201/eid2306.160120)
Supplement: Technical Appendix — Baseline characteristics of Middle East respiratory syndrome case-patients, Daejeon, South Korea, 2015. [file 16-0120-Techapp-s1.pdf]

Article DOI: http://dx.doi.org/10.3201/eid2306.160120

Hospital Outbreaks of Middle East Respiratory Syndrome, Daejeon, South Korea, 2015

Technical Appendix

Technical Appendix Table. Baseline characteristics of Middle East respiratory syndrome case-patients, Daejeon, South Korea, 2015*

Case No.†	Age	Sex	Role	Underlying disease	Smoking	Symptoms at diagnosis	Ambulation status	Hospital	Outcome
16	41	Male	Patient	FAP	Ex-smoker	Fever, chills, cough, sputum, myalgia, dyspnea, nausea, diarrhea, sore throat	No limitation	A, B	Recovery
23	73	Male	Patient	HTN, COPD	Current smoker	Fever, chills, sputum, dyspnea, anorexia	Total bed rest	B	Death
24	78	Male	Patient	HTN, CHF, COPD, asthma prostate Ca	Non-smoker	Fever, chills, cough, sputum, dyspnea	Total bed rest	B	Death
30	60	Male	Patient	None	Non-smoker	Fever, chills, myalgia, sore throat, rhinorrhea	No limitation	A	Recovery
31	69	Male	Patient	HTN, pul TB, mesenteric thrombosis	Ex- smoker	Fever, chills, cough, sputum, hemoptysis, myalgia, nausea	Isolated because of pul TB	B	Death
36	82	Male	Patient	HTN, asthma	Ex-smoker	Fever, dyspnea	Active movement with wheelchair	B	Death
38	49	Male	Patient	HTN, DM, alcoholic LC	Current smoker	Fever, myalgia, headache, diarrhea	Active movement with wheelchair	A	Death
45	64	Male	Patient	None	Ex-smoker	Fever	No limitation	B	Death
54	63	Female	Caregiver	None	Non-smoker	Fever, chills, headache	No limitation	A	Recovery
82	82	Female	Family caregiver	HTN	Non-smoker	Fever, cough, sputum	No limitation	B	Death
83	64	Male	Patient	Lung Ca colon Ca	Ex-smoker	Fever	Total bed rest	B	Death
84	81	Male	Patient	Stomach Ca, DI	Non-smoker	Fever, sputum, myalgia, dyspnea, nausea	Total bed rest	A	Death
85	66	Female	Caregiver	None	Non-smoker	Fever	No limitation	A	Recovery
86	76	Female	Patient	HTN, DM dementia	Non-smoker	Fever, dyspnea, diarrhea	Total bed rest	A	Recovery
87	78	Female	Patient	HTN, dementia Parkinson’s disease	Non-smoker	Fever	Total bed rest	A	Death
95	76	Male	Patient	HTN, lung Ca	Ex-smoker	Fever	No limitation	B	Recovery
106	60	Female	Caregiver	HTN, DM	-	Fever, chills, cough, myalgia	No limitation	B	Recovery
107	64	Female	Caregiver	None	Non-smoker	Fever	No limitation	A	Recovery
127	75	Female	Family caregiver	HTN	-	Fever, chills, myalgia	No limitation	B	Recovery

Case No.†	Age	Sex	Role	Underlying disease	Smoking	Symptoms at diagnosis	Ambulation status	Hospital	Outcome
128	87	Male	Patient	HTN<br>CVD	Non-smoker	Fever, cough, rhinorrhea	Passive movement with wheelchair	A	Death
129	86	Male	Patient	HTN<br>DM<br>dementia<br>Parkinson's disease<br>IPF	Non-smoker	Fever, diarrhea	Passive movement with wheelchair	A	Recovery
130	65	Female	Caregiver	DM	Non-smoker	Fever, cough	No limitation	A	Recovery
143	31	Male	Engineer	None	Non-smoker	Febrile sensation with abdominal discomfort	No limitation	A	Recovery
148	38	Female	Nurse	None	Non-smoker	Fever, chills, myalgia, headache, dizziness	No limitation	B	Recovery
149	84	Female	Patient	HTN<br>DM	Non-smoker	Fever, vomiting, diarrhea	Passive movement with wheelchair	A	Recovery
172	61	Female	Caregiver	CVD	-	Fever, chills, diarrhea	No limitation	A	Recovery

*Ca, Cancer; CHF, Congestive heart failure; COPD, Chronic obstructive pulmonary disease; CVD, Cerebrovascular disease; DI, Diabetes insipidus, DM, Diabetes mellitus; FAP, Familial adenomatous polyposis; HTN, Hypertension; IPF, Idiopathic pulmonary fibrosis; LC, Liver cirrhosis; Pul TB, Pulmonary tuberculosis.

†Each case number refers to the order in which the case was confirmed in the context of all cases reported during the MERS outbreak in South Korea.
